# Remodeling of Human Osteochondral Defects via rAAV-Mediated Co-Overexpression of TGF-β and IGF-I from Implanted Human Bone Marrow-Derived Mesenchymal Stromal Cells

**DOI:** 10.3390/jcm8091326

**Published:** 2019-08-28

**Authors:** Stephanie Morscheid, Jagadeesh Kumar Venkatesan, Ana Rey-Rico, Gertrud Schmitt, Magali Cucchiarini

**Affiliations:** Center of Experimental Orthopaedics, Saarland University Medical Center and Saarland University, 66421 Homburg/Saar, Germany

**Keywords:** osteochondral defect repair, hMSCs, rAAV vectors, TGF-β, IGF-I

## Abstract

The application of chondrogenic gene sequences to human bone marrow-derived mesenchymal stromal cells (hMSCs) is an attractive strategy to activate the reparative activities of these cells as a means to enhance the processes of cartilage repair using indirect cell transplantation procedures that may improve the repopulation of cartilage lesions. In the present study, we examined the feasibility of co-delivering the highly competent transforming growth factor beta (TGF-β) with the insulin-like growth factor I (IGF-I) in hMSCs via recombinant adeno-associated virus (rAAV) vector-mediated gene transfer prior to implantation in a human model of osteochondral defect (OCD) ex vivo that provides a microenvironment similar to that of focal cartilage lesions. The successful co-overexpression of rAAV TGF-β/IGF-I in implanted hMSCs promoted the durable remodeling of tissue injury in human OCDs over a prolonged period of time (21 days) relative to individual gene transfer and the control (reporter *lac*Z gene) treatment, with enhanced levels of cell proliferation and matrix deposition (proteoglycans, type-II collagen) both in the lesions and at a distance, while hypertrophic, osteogenic, and catabolic processes could be advantageously delayed. These findings demonstrate the value of indirect, progenitor cell-based combined rAAV gene therapy to treat human focal cartilage defects in a natural environment as a basis for future clinical applications.

## 1. Introduction

Focal articular cartilage lesions remain a particularly challenging clinical problem in orthopaedics due to the failure of the articular chondrocytes, the only cell population present in this highly specialized, avascular tissue, to fully provide self-repair activities in sites of cartilage injury [1,2,3,4]. Equally critical, none of the surgical options currently employed in orthopaedic surgery to mobilize or even supply chondroregenerative progenitor cells, like those from the subchondral bone marrow (microfracture, pridie drilling, stromal cell transplantation), can reliably support the strict reproduction of the original hyaline cartilage in such lesions with a typical proteoglycan- and type-II collagen-based extracellular matrix (ECM) [1,2,3,4,5,6,7]. Instead, a fibrocartilaginous repair tissue mostly composed of type-I collagen is generated in sites of cartilage injury, without the proper mechanical integrity that would allow to withstand mechanical loads over time, and that may progress towards irreversible, degenerative osteoarthritis [1,2,3,4].

Strategies that aim to activate the chondroreparative properties of progenitor cells prior to reimplantation in sites of cartilage damage may provide strong tools to overcome these hurdles, like approaches using gene therapy procedures that support the specific and long-term targeting of cellular and biological pathways involved in cartilage repair [8,9]. In this regard, vectors derived from the human adeno-associated virus (AAV) vector are particularly well suited to achieve these goals from a translational perspective [10], as recombinant AAV (rAAV) constructs can safely and durably transduce bone marrow-derived mesenchymal stromal cells (MSCs) at very high efficiencies (about 100% for several weeks) [11,12,13,14,15,16,17]. While the rAAV-mediated transduction of MSCs from human patients (hMSCs) has been attempted in a number of trials in vitro by delivering chondrotherapeutic genes coding for the basic fibroblast growth factor (FGF-2) [13], transforming growth factor beta 1 (TGF-β1) [11,15], insulin-like growth factor I (IGF-I) [16], sex-determining region Y-type high-mobility group box 9 transcription factor (SOX9) [14,17], little is known on the benefits of rAAV for the application of genetically modified hMSCs in human focal cartilage defects in a native microenvironment.

In the present study, we focused on co-delivering TGF-β and IGF-I, two potent factors supporting cartilage repair, in hMSCs via rAAV as improved cell regenerative platforms capable of enhancing the repair activities in a human osteochondral defect (OCD) model system ex vivo, and, in light of our previous work in vitro, showing the feasibility of co-applying these two candidates in settings aiming at directly rejuvenating resident MSCs within a lesion [18]. For the first time, and to our best knowledge, the current work demonstrates that the simultaneous overexpression of TGF-β and IGF-I can be effectively achieved in rAAV-targeted hMSCs implanted within human OCDs, promoting the sustained remodeling of the injured human articular cartilage (cell proliferation, hyaline ECM deposition) over a prolonged period of 21 days while containing unwanted hypertrophic, osteogenic, and catabolic processes relative to independent gene treatments and control condition (reporter *lac*Z gene transfer). These findings show the value of using combined cell- and gene-based therapy via rAAV as a significant means to enhance human cartilage repair in environmental conditions closely resembling those in patients in vivo.

## 2. Experimental Section

### 2.1. Study Design

**Figure 1:**
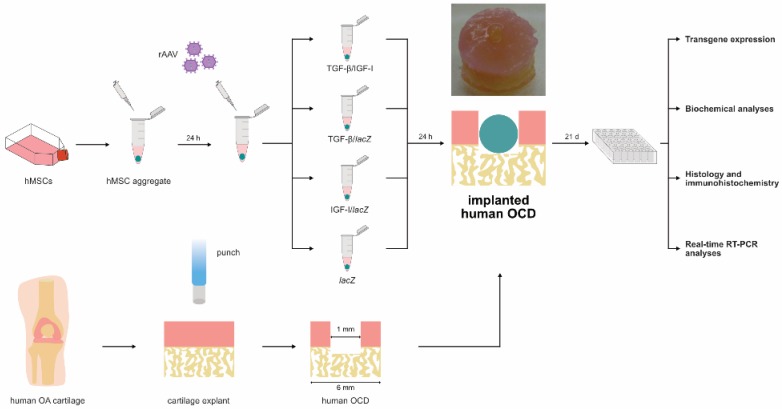
Study design. Human bone marrow-derived mesenchymal stromal cells MSCs were prepared as high-density aggregates (2 × 10^5^ cells/aggregate) in defined chondrogenic medium overnight, transduced with the various rAAV shuttles (TGF-β/IGF-I, TGF-β/*lac*Z, IGF-I/*lac*Z: 40 µL each vector/aggregate; *lacZ*: 80 µL vector/aggregate), and directly applied to human osteochondral defects (OCDs; 6-mm diameter) for further culture in defined chondrogenic medium for 21 days when the OCDs were processed to monitor transgene expression and for biochemical, immunohistochemical/histological, and real-time RT-PCR.

Human bone marrow-derived MSCs were prepared as high-density aggregates in defined chondrogenic medium and subsequently transduced with the various rAAV vectors (total MOI = 8) as candidate combined rAAV-hTGF-β/rAAV-hIGF-I (TGF-β/IGF-I; 40 µL each vector/aggregate) versus independent rAAV-hTGF-β (TGF-β/*lac*Z) or rAAV-hIGF-I (IGF-I/*lac*Z) gene transfer (40 µL each vector/aggregate) or absence of therapeutic gene (reporter rAAV-*lac*Z vector, i.e., *lac*Z condition; 80 µL vector/aggregate). Aggregates were then implanted after 24 h in experimentally created human osteochondral defects (OCDs; 1 aggregate/OCD) in a patient-match manner when both samples were available that were maintained in defined chondrogenic medium for 21 days [5] to evaluate transgene expression and to establish biochemical assays, immunohistochemical and histological analyses, and real-time RT-PCR analyses (Figure 1).

### 2.2. Reagents

All reagents were purchased from Sigma, Munich (Germany) unless otherwise indicated. The dimethylmethylene blue dye (DMMB) was from Serva, Heidelberg (Germany). The anti-TGF-β (V) antibody was purchased at Santa Cruz Biotechnology (Heidelberg, Germany), the anti-IGF-I (AF-291-NA) at R&D Systems (Wiesbaden, Germany), the anti-type-II collagen (II-II6B3) antibody at NIH Hybridome Bank (university of Iowa, Ames, IA, USA), the anti-type-I collagen (AF-5610) antibody at Acris Antibodies, Hiddenhausen (Germany), and the anti-type-X collagen (COL10) antibody at Sigma. Biotinylated secondary antibodies and ABC reagent were from Vector Laboratories Alexis Deutschland, Grünberg (Germany). The TGF-β and IGF-I Quantikine ELISAs were purchased at R&D Systems and the AAVanced Concentration Reagent at System Bioscience (Heidelberg, Germany).

### 2.3. Cell and Osteochondral Defect Cultures

Bone marrow aspirates were collected from the distal femurs of patients undergoing total knee arthroplasty (*n* = 3) with informed consent and according to the Helsinki Declaration. The study was approved by the Ethics Committee of the Saarland Physicians Council (No. 270-17). Firstly, bone marrow-derived hMSCs were isolated and expanded as previously described [13,14,15,16,17,18,19]. Briefly, freshly isolated hMSCs were washed, centrifuged in Dulbecco’s modified Eagle’s medium (DMEM) and the pellet obtained was resuspended in a mixture of red blood cell lysing buffer and DMEM in equal ratios. The mixture was washed, pelleted, and resuspended in DMEM containing 10% fetal bovine serum with 100 U/mL penicillin and 100 µL/mL streptomycin. Cells were plated in T75 flasks and kept incubated overnight at 37 °C under 5% CO_2_, and the medium was removed and replaced by growth medium with recombinant FGF-2 (1 ng/mL), with a medium change every 2–3 days. Cells were tested at passages 1–2 in order to avoid a shift in cell phenotype.

Human osteoarthritic (OA) cartilage biopsies excluding unaffected and fibrocartilage (*n* = 9; 6-mm diameter; Mankin score = 7–9) randomly collected from the femoral condyle of patients undergoing total knee arthroplasty were used to create standardized osteochondral defects (OCDs) with a 1-mm biopsy punch [20,21] and briefly kept in growth medium for 2–3 days (to avoid a shift in cell phenotype in the tissue) prior to direct implantation of the aggregates.

### 2.4. Plasmids and rAAV Vectors

All vectors were produced from the AAV-2-based vector plasmid pSSV9 [22,23]. It is known that rAAV-*lac*Z carries the *lac*Z gene encoding the *E. coli* β-galactosidase (β-gal). Further, rAAV-hTGF-β carries a human transforming growth factor beta 1 (hTGF-β) cDNA and rAAV-hIGF-I, a human insulin-like growth factor I (hIGF-I) cDNA, both clones instead of *lacZ* in rAAV-*lac*Z, and all genes were controlled by the cytomegalovirus immediate-early (CMV-IE) promoter/enhancer [13,14,15,16,17,21,24]. Subsequently, rAAV vectors were prepared as conventional (not self-complementary) vectors using a helper-based system maintaining Adenovirus 5 and Ad8 in the 293 cell line (an adenovirus transformed human embryonic kidney packaging cell line) [13,14,15,16,17,21,24]. The vector preparations were purified by dialysis using the AAVanced Concentration Reagent and titrated by real-time PCR as previously described (approximately 10^10^ transgene copies/mL and ratio recombinant functional viral particles to recombinant viral particles of about 1/500) [13,14,15,16,17,21,24].

### 2.5. rAAV-Mediated Gene Transfer

Bone marrow-derived hMSCs were prepared as high-density cultures (2 × 10^5^ cells/aggregate) and incubated in 150 µL chondrogenic medium (DMEM high glucose 4.5 g/L, ITS^+^ Premix, 1 mM pyruvate, ascorbate 2-phosphate 37.5 ng/mL, 10^−7^ M dexamethasone, TGF-β3 10 ng/mL) overnight at 37 °C under 5% CO_2_ prior to vector addition [13,14,15,16,17,21]. Aggregates were then directly transduced with the vector treatments (TGF-β/IGF-I, TGF-β/*lac*Z, or IGF-I/*lac*Z: 40 µL each vector/aggregate; *lac*Z: 80 µL vector/aggregate; total MOI = 8) and 150 μL chondrogenic medium was added for incubation at 37 °C under 5% CO_2_ for 24 h. The (co-)transduced aggregates were then directly applied to the OCDs, with incubation in 750 µL chondrogenic medium for 21 days. The chondrogenic medium was exchanged every 2–3 days.

### 2.6. Transgene Expression

The expression of the candidate TGF-β and IGF-I gene products was assessed by specific ELISAs. Briefly, the OCDs were washed twice with serum-free medium and kept in 750 µL DMEM for 24 h. Culture supernatants were then collected and centrifuged and tested following the manufacturer’s recommendations on a GENios spectrophotometer/fluorometer (Tecan, Crailsheim, Germany) [15,16,17,24].

### 2.7. Histological and Immonohistochemical Analyses

The OCDs were harvested after 21 days, fixed in formaldehyde (4%), washed with PBS, decalcified in 10% sodium citrate, 25% formic acid for two weeks at room temperature, and dehydrated in graded alcohol [21]. Histological and immunohistochemical analyses were performed on paraffin-embedded sections of OCDs (10 µm). Sections were stained with hematoxylin and eosin (H&E) (cellularization) and safranin O (matrix proteoglycans) [21]. The expression of type-II, -I, and -X collagen was monitored by immunohistochemical detection using specific primary antibodies, biotinylated secondary antibodies, and the ABC method with diaminobenzidine (DAB) as a chromogen [21]. Samples were analyzed under light microscopy (Olympus BX 45, Hamburg, Germany).

### 2.8. Histomorphometric Analyses

The transduction efficiencies (% of TGF-β^+^ or IGF-I^+^ immunoreactive cells to the total cell number), the cell densities (cells/mm^2^), and the intensities of safranin O staining and those of type-II, -I, and -X collagen immunostaining (ratio tissue surface showing positive immunoreactivity for particular collagen/total tissue surface) were measured using the SIS analysisSIS program (Olympus), Adobe photoshop (Adobe Systems, Unterschleissheim, Germany), Scion Image (Scion Corporation, Frederick, MD, USA) at three random sites standardized for their surface or using serial histological or immunohistochemical sections [21]. Sections were also scored to evaluate the cartilage surrounding the OCDs (magnification × 10) and the implanted aggregates (magnification × 20) for uniformity and intensity using a modified Bern Score grading system [19,21]: 0 (no staining), 1 (heterogenous and/or weak staining), 2 (homogeneous and/or moderate staining), 3 (homogeneous and/or intense staining), 4 (very intense staining).

### 2.9. Biochemical Analyses

The OCDs were collected after 21 days of culture and digested in papain to determine the DNA contents by using the Hoechst 33,258 fluorometric assay, the proteoglycan contents by binding to the dimethylmethylene blue dye (DMMB), and the total cellular protein contents for normalization with the Pierce protein assay (Pierce Thermo Scientific, Fisher Scientific, Schwerte, Germany) [21]. Measurements were performed with a GENios spectrophotometer/fluorometer (Tecan).

### 2.10. Real-Time RT-PCR Analyses

Total RNA was extracted from the OCDs containing the aggregates but excluding bone tissue (RNeasy Protect Mini kit, Qiagen, Hilden, Germany) for reverse transcription (1st Strand cDNA Synthesis kit, avian myeloblastis virus (AMV) (Roche Applied Science, Mannheim, Germany). cDNA amplification was performed via SYBR Green real-time RT-PCR (Stratagene, Agilent Technologies, Waldbronn, Germany) maintaining initial incubation (95 °C, 10 min), 55 cycles of amplification (denaturation (95 °C, 30 s), annealing (55 °C, 1 min), extension (72 °C, 30 s), denaturation (95 °C 1 min), and final incubation (55 °C, 30 s). The primers employed were: SOX9 (chondrogenic marker) (forward 5′-ACACACAGCTCACTCGACCTTG-3′; reverse 5′-GGGAATTCTGGTTGGTCCTCT-3′), type-II collagen (COL2A1, chondrogenic marker) (forward 5′-GGACTTTTCTCCCCTCTCT-3; reverse 5′-GACCCGAAGGTCTTACAGGA-3′), aggrecan (ACAN, chondrogenic marker) (forward 5′-GAGATGGAGGGTGAGGTC-3′; reverse 5′-ACGCTGCCTCGGGCTTC-3′), type-I collagen (COL1A1, fibrocartilage and osteogenic marker) (forward 5′-ACGTCCTGGTGAAGTTGGTC-3′; reverse 5′-ACCAGGGAAGCCTCTCTCTC-3′), type-X collagen (COL10A1, hypertrophic marker) (forward 5′-CCCTCTTGTTAGTGCCAACC-3′; reverse 5′-AGATTCCAGTCCTTGGGTCA-3′), matrix metalloproteinase 13 (MMP13, marker of terminal differentiation and catabolism) (forward 5′- AATTTTCACTTTTGGCAATGA-3′; reverse 5′-CAAATAATTTATGAAAAAGGGATGC-3′), and glyceraldehyde-3-phosphate dehydrogenase (GAPDH) (housekeeping gene and internal control) (forward 5′-GAAGGTGAAGGTCGGAGTC-3′; reverse 5′-GAAGATGGTGATGGGATTTC-3′) [13,14,15,16,17]. Control conditions included reactions using water and non-reverse-transcribed mRNA. The specificity of the products was confirmed by melting curve analysis and agarose gel electrophoresis. The threshold cycle (Ct) value for each gene of interest was measured for each amplified sample using MxPro QPCR software (Stratagene), and values were normalized to GAPDH expression by using the 2^−ΔΔCt^ method [13,14,15,16,17].

### 2.11. Statistical Analysis

Each condition was performed in duplicate in three independent experiments for each patient. All hMSCs aggregates were tested for all experiments using the human OCDs. Results are expressed as the mean ± standard deviation (SD). A t-test was employed with *p* < 0.05 considered statistically significant.

## 3. Results

### 3.1. Effective rAAV-Mediated Co-Overexpression of TGF-β and IGF-I in Human Osteochondral Defects Upon Implantation of Genetically Modified hMSC Aggregates

**Figure 2:**
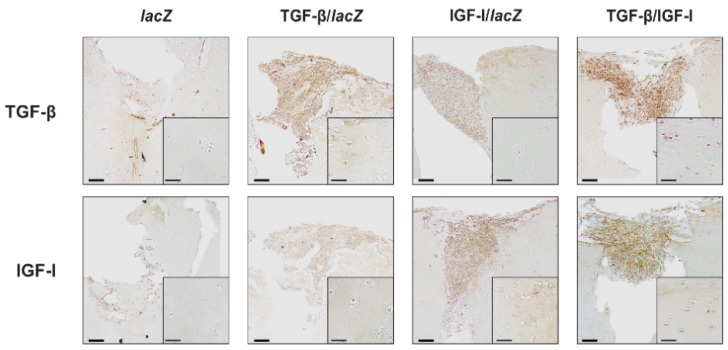
Detection of transgene (TGF-β, IGF-I) overexpression in human OCDs following implantation of rAAV-transduced hMSC aggregates. Aggregates were cotransduced with rAAV TGF-β/IGF-I, TGF-β/*lac*Z, IGF-I/*lac*Z or transduced with rAAV *lacZ* and implanted in human OCDs as described in Figure 1 and in the Materials and Methods. Samples were processed after 21 days to detect the expression of TGF-β and of IGF-I by immunohistochemistry (magnification ×10; insets showing the surrounding cartilage in the region immediately adjacent to the implanted pellets at magnification × 20; representative data). Scale bars: 100 µm, insets: 50 µm.

The rAAV vectors were first applied to hMSC aggregates (*n* = 3) and (co-)transduced aggregates were then directly implanted in human OCDs (*n* = 3) according to the study design (Figure 1) to test the ability of rAAV to co-overexpress the therapeutic TGF-β and IGF-I candidates (TGF-β/IGF-I) over time (21 days) versus independent gene transfer (TGF-β/*lac*Z or IGF-I/*lac*Z) and control (*lac*Z) condition (endogenous growth factor expression). Effective expression of TGF-β and IGF-I via rAAV was noted in the specific conditions examined, especially in the TGF-β/IGF-I OCDs (Table 1, Table 2 and Figure 2).

The levels of TGF-β production were higher in the TGF-β/IGF-I than in the *lac*Z, TGF-β/*lac*Z, and IGF-I/*lac*Z OCDs (3.6-, 1.7-, and 3.5-fold difference, respectively, *p* ≤ 0.001) and in the TGF-β/*lac*Z versus *lac*Z and IGF-I/*lac*Z OCDs (2.1-fold difference, *p* ≤ 0.010) while there was no difference between the IGF-I/*lac*Z and *lac*Z OCDs (*p* = 0.500) (Table 1). The levels of IGF-I production were higher in the TGF-β/IGF-I than in the *lac*Z, TGF-β/*lac*Z, and IGF-I/*lac*Z OCDs (1.4-, 1.3-, and 1.1-fold difference, *p* = 0.010, *p* = 0.010, and *p* = 0.430, respectively) and in the IGF-I/*lac*Z versus *lac*Z and TGF-β/*lac*Z OCDs (1.3-fold difference, *p* ≤ 0.040) while there was no difference between the TGF-β/*lac*Z and *lac*Z OCDs (*p* = 0.353) (Table 1).

Immunoreactivity to TGF-β was higher in the TGF-β/IGF-I than in the *lacZ*, TGF-β/*lac*Z, and IGF-I/*lac*Z OCDs (7.8-, 1.2-, and 7.8-fold difference, respectively, *p* ≤ 0.020) and in the TGF-β/*lac*Z versus *lac*Z and IGF-I/*lac*Z OCDs (6.4-fold difference, *p* ≤ 0.001) while there was no difference between the IGF-I/*lac*Z and *lac*Z OCDs (*p* = 0.500) (Figure 2 and Table 2). In the surrounding cartilage, TGF-β immunoreactivity was higher in the TGF-β/IGF-I than in the *lac*Z, TGF-β/*lac*Z, and IGF-I/*lac*Z samples (37-, 1.2-, and 24.7-fold difference, respectively, *p* ≤ 0.040) and in the TGF-β/*lac*Z versus *lac*Z and IGF-I/*lac*Z samples (31- and 20.7-fold difference, respectively, *p* = 0.005) while there was no difference between the IGF-I/*lac*Z and *lac*Z samples (1.5-fold difference, *p* = 0.220) (Figure 2 and Table 2). Immunoreactivity to IGF-I was higher in the TGF-β/IGF-I than in the *lac*Z, TGF-β/*lac*Z, and IGF-I/*lac*Z OCDs (6.6-, 4.8-, and 1.2-fold difference, respectively, *p* ≤ 0.002) and in the IGF-I/*lac*Z versus *lac*Z and TGF-β/*lac*Z OCDs (5.8 and 4.2-fold difference, respectively, *p* ≤ 0.001) while there was no difference between the TGF-β/*lac*Z versus *lac*Z OCDs (*p* = 0.077) (Figure 2 and Table 2). In the surrounding cartilage, IGF-I immunoreactivity was higher in the TGF-β/IGF-I than in the *lac*Z, TGF-β/*lac*Z, and IGF-I/*lac*Z samples (10.7-, 4.3-, and 1.2-fold difference, respectively, *p* ≤ 0.041) and in the IGF-I/*lac*Z versus *lac*Z and TGF-β/*lac*Z samples (9.3- and 3.7-fold difference, respectively, *p* ≤ 0.020) while there was no difference between the TGF-β/*lac*Z versus *lac*Z samples (*p* = 0.240) (Figure 2 and Table 2).

### 3.2. Biological and Chondrogenic Differentiation Activities in Human Osteochondral Defects Mediated by Co-Overexpression of TGF-β and IGF-I via rAAV in Implanted hMSC Aggregates

The hMSC aggregates (*n* = 3) transduced with the various rAAV vectors were next directly implanted in human OCDs (*n* = 3) to determine the capacity of the TGF-β/IGF-I combination to enhance the proliferative, anabolic, and chondrogenic activities in sites of transplantation in situ over time (21 days) versus independent gene (TGF-β/*lac*Z or IGF-I/*lac*Z) and control (*lac*Z) gene treatments.

The DNA contents were higher in the TGF-β/IGF-I versus *lac*Z, TGF-β/*lac*Z, and IGF-I/*lac*Z OCDs (1.3-, 1.2-, and 1.3-fold difference, respectively, *p* ≤ 0.020) and in the TGF-β/*lac*Z versus *lacZ* and IGF-I/*lac*Z OCDs (1.2-fold difference, *p* ≥ 0.060) while there was no difference between the IGF-I/*lac*Z and *lac*Z OCDs (*p* = 0.440) (Table 3).

**Figure 3:**
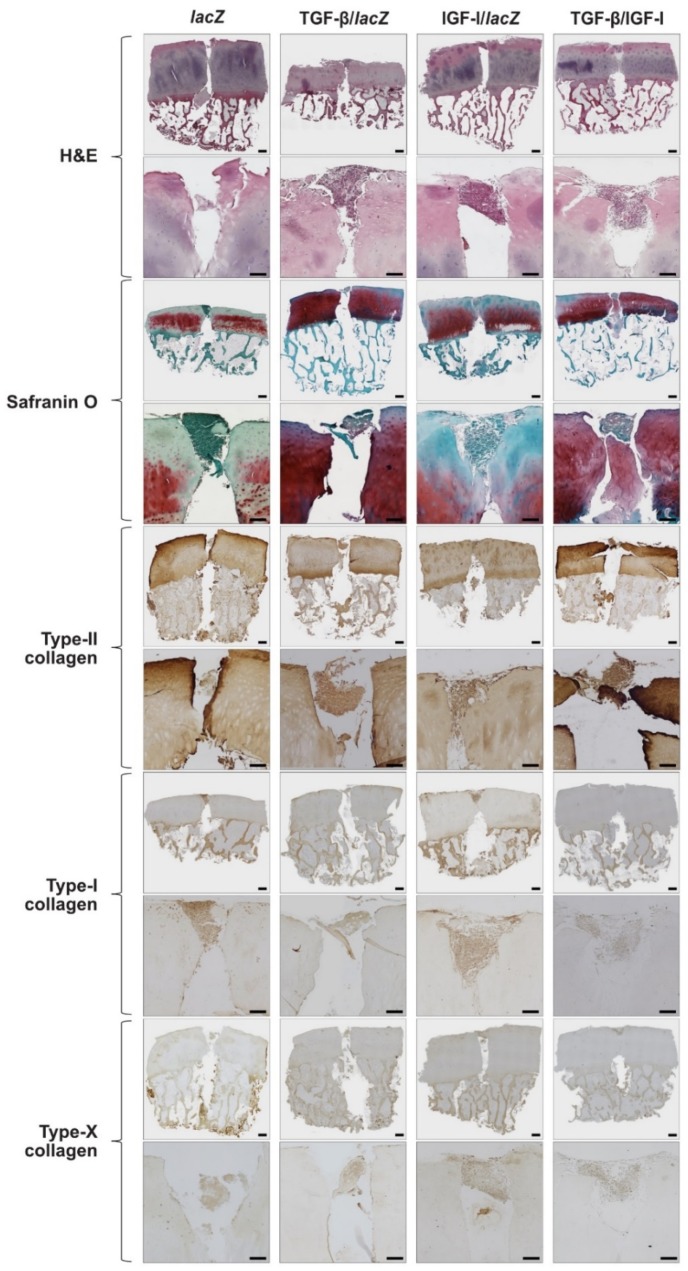
Biological, chondrogenic, osteogenic, and hypertrophic differentiation activities in human OCDs following implantation of rAAV-transduced hMSC aggregates. Aggregates were cotransduced with rAAV TGF-β/IGF-I, TGF-β/*lac*Z, IGF-I/*lac*Z or transduced with rAAV *lac*Z and implanted in human OCDs as described in Figure 1 and Figure 2 and in the Materials and Methods. Samples were processed after 21 days to evaluate cellularity (H&E staining) and the deposition of matrix proteoglycans (safranin O staining), type-II/-I/-X collagen (immunohistochemistry) (for each type of staining: top panel at magnification × 10 with scale bars of 500 µm, bottom panels including the region immediately adjacent to the implanted pellets at magnification ×20 with scale bars of 200 µm; representative data).

The cell densities were higher in the TGF-β/IGF-I versus *lac*Z, TGF-β/*lac*Z, and IGF-I/*lac*Z OCDs (3.2-, 2-, and 2-fold difference, respectively, *p* ≤ 0.020), in the TGF-β/*lac*Z versus *lac*Z OCDs (1.7-fold difference, *p* = 0.044), and in the IGF-I/*lac*Z versus *lac*Z OCDs (1.6-fold difference, *p* = 0.006) while there was no difference between the TGF-β/*lac*Z and IGF-I/*lac*Z OCDs (*p* = 0.390) (Figure 3 and Table 2). In the cartilage surrounding the OCDs, the cell densities were higher in the TGF-β/IGF-I versus *lac*Z, TGF-β/*lac*Z, and IGF-I/*lac*Z samples (2-, 1.2-, and 1.7-fold difference, respectively, *p* ≤ 0.040), in the TGF-β/*lac*Z versus *lac*Z and IGF-I/*lac*Z samples (1.6- and 1.4-fold difference, respectively, *p* ≤ 0.040), and in the IGF-I/*lac*Z and *lac*Z samples (1.2-fold difference, *p* = 0.090) (Figure 3 and Table 2).

The anabolic and chondrogenic differentiation activities were higher in the TGF-β/IGF-I versus *lac*Z, TGF-β/*lac*Z, and IGF-I/*lac*Z OCDs (proteoglycans: 1.8-, 1.3-, and 1.5-fold difference, respectively, *p* ≤ 0.020; proteoglycans/DNA: 1.4-, 1.2-, and 1.3-fold difference, respectively, *p* ≤ 0.030; safranin O: 8-, 1.1-, and 24-fold difference, *p* ≤ 0.001, *p* = 0.260, and *p* ≤ 0.001, respectively; type-II collagen: 3.8-, and 1.7-fold difference, respectively, *p* ≤ 0.030), in the TGF-β/*lac*Z versus *lac*Z and IGF-I/*lac*Z OCDs (proteoglycans: 1.4- and 1.2-fold difference, *p* = 0.030 and *p* = 0.200, respectively; proteoglycans/DNA: 1.2- and 1.1-fold difference, respectively, *p* = 0.030 and *p* = 0.150; safranin O: 7- and 21-fold difference, respectively, *p* ≤ 0.001; type-II collagen: 3- and 1.3-fold difference, respectively, *p* ≤ 0.030), and in the IGF-I/*lac*Z versus *lac*Z OCDs (proteoglycans: 1.3-fold difference, *p* = 0.090; proteoglycans/DNA: 1.1-fold difference, *p* = 0.280; type-II collagen: 2.3-fold difference, *p* = 0.010) while there was no difference between the IGF-I/*lac*Z and *lac*Z OCDs for safranin O staining (*p* = 0.500) (Table 2, Table 3 and Figure 3). In the cartilage surrounding the OCDs, the chondrogenic differentiation activities were higher in the TGF-β/IGF-I versus *lac*Z, TGF-β/*lac*Z, and IGF-I/*lac*Z samples (safranin O: 2.9-, 1.5-, and 2-fold difference, respectively, *p* ≤ 0.030; type-II collagen: 6-, 1.2-, and 1.7-fold difference, *p* = 0.002, *p* = 0.100, and *p* = 0.040, respectively), in the TGF-β/*lac*Z versus *lac*Z and IGF-I/*lac*Z samples (safranin O: 2- and 1.4-fold difference, *p* = 0.010 and p = 0.080, respectively; type-II collagen: 5- and 1.4-fold difference, respectively, *p* ≤ 0.030), and in the IGF-I/*lac*Z versus *lac*Z samples (safranin O: 1.5-fold difference, *p* ≤ 0.030; type-II collagen: 3.6-fold difference, *p* = 0.040) (Table 2, Table 3 and Figure 3).

### 3.3. Influence of Co-Overexpression of TGF-β and IGF-I via rAAV Upon the Osteogenic and Hypertrophic Differentiation Activities in Human Osteochondral Defects Upon Implantation of Genetically Modified hMSC Aggregates

The hMSC aggregates (*n* = 3) transduced with rAAV were then directly implanted in human OCDs (*n* = 3) to determine the potential influence of the TGF-β/IGF-I combination on the osteogenic and hypertrophic activities in transplanted sites in situ over time (21 days) versus independent gene delivery (TGF-β/*lac*Z or IGF-I/*lac*Z) and control (*lac*Z) treatment.

The osteogenic differentiation activities (type-I collagen deposition) were lower in the TGF-β/IGF-I versus *lac*Z, TGF-β/*lac*Z, and IGF-I/*lac*Z OCDs (2.8-, 1.4-, and 2.7-fold difference, *p* ≤ 0.001, *p* = 0.090, and *p* ≤ 0.001, respectively) and in the TGF-β/*lac*Z versus *lac*Z and IGF-I/*lac*Z OCDs (2.1- and 2-fold difference, respectively, *p* ≤ 0.001) while there was no difference between the IGF-I/*lac*Z and *lac*Z OCDs (*p* = 0.400) (Figure 3 and Table 2). In the cartilage surrounding the OCDs, the osteogenic differentiation activities were lower in the TGF-β/IGF-I versus *lac*Z, TGF-β/*lac*Z, and IGF-I/*lac*Z samples (1.9-, 1.3-, and 1.7-fold difference, *p* ≤ 0.001, *p* = 0.120, and *p* ≤ 0.001, respectively), in the TGF-β/*lac*Z versus *lac*Z and IGF-I/*lac*Z samples (1.5- and 1.3-fold difference, *p* = 0.020 and *p* = 0.130, respectively), and in the IGF-I/*lac*Z and *lac*Z samples (1.1-fold difference, *p* = 0.230) (Figure 3 and Table 2).

The hypertrophic differentiation activities (type-X collagen deposition) were lower in the TGF-β/IGF-I versus *lac*Z, TGF-β/*lac*Z, and IGF-I/*lac*Z OCDs (2.3-, 1.5-, and 1.8-fold difference, *p* ≤ 0.001, *p* = 0.100, and *p* = 0.100, respectively), in the TGF-β/*lac*Z versus *lac*Z and IGF-I/*lac*Z OCDs (1.6- and 1.2-fold difference, respectively, *p* ≤ 0.001 and *p* = 0.260, respectively), and in the IGF-I/*lac*Z versus *lac*Z OCDs (1.3-fold difference, *p* = 0.090) (Figure 3 and Table 2). In the cartilage surrounding the OCDs, the hypertrophic differentiation activities were lower in the TGF-β/IGF-I versus *lac*Z, TGF-β/*lac*Z, and IGF-I/*lac*Z samples (3.6-, 3.3-, and 2.6-fold difference, respectively, *p* ≤ 0.007), in the TGF-β/*lacZ* versus *lac*Z samples (1.1-fold difference, *p* = 0.230), in the IGF-I/*lac*Z versus samples (1.4-fold difference, *p* = 0.060), and and in the IGF-I/*lac*Z versus TGF-β/*lac*Z samples (1.2-fold difference, *p* = 0.230) (Figure 3 and Table 2).

### 3.4. Real-Time RT-PCR Analyses in Human Osteochondral Defects Mediated by Co-Overexpression of TGF-β with IGF-I via rAAV in Implanted hMSC Aggregates

**Figure 4:**
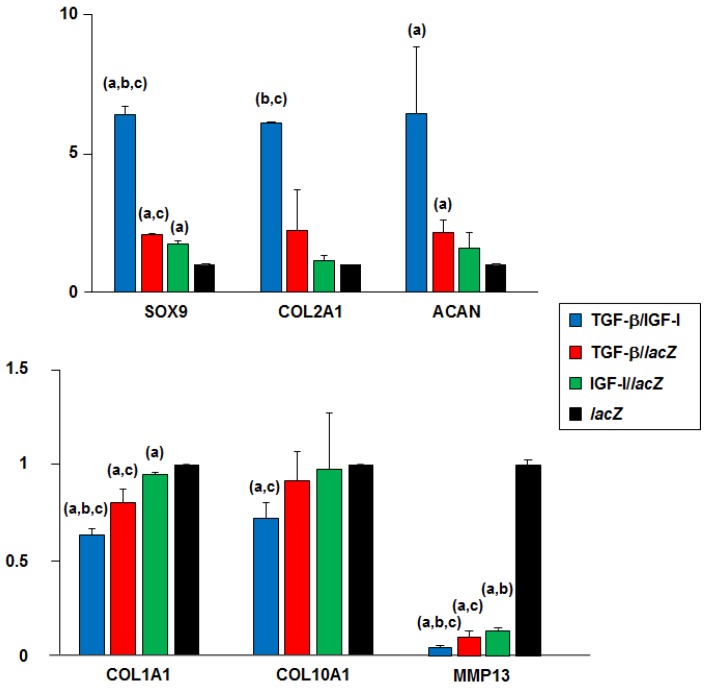
Real-time RT-PCR analyses in human OCDs following implantation of rAAV-transduced hMSC aggregates. Aggregates were cotransduced with rAAV TGF-β/IGF-I, TGF-β/*lacZ*, IGF-I/*lacZ* or transduced with rAAV *lacZ* and implanted in human OCDs as described in Figure 1, Figure 2 and Figure 3 and in the Materials and Methods. Samples were processed after 21 days to monitor the gene expression profiles by real-time RT-PCR as described in the Materials and Methods. The genes analyzed included the transcription factor SOX9, type-II collagen (COL2A1), aggrecan (ACAN), type-I collagen (COL1A1), type-X collagen (COL10A1), and matrix metalloproteinase 13 (MMP13), with GAPDH serving as a housekeeping gene and internal control. Threshold cycle (Ct) values were obtained for each target and GAPDH as a control for normalization, and fold inductions (relative to OCDs where *lacZ* aggregates were implanted) were measured using the 2^−ΔΔCt^ method. Statistically significant relative to ^a^*lacZ*, ^b^TGF-β/*lacZ*, and ^c^IGF-I/*lacZ*.

These results were corroborated by real-time RT-PCR analysis in human OCDs (*n* = 3) where rAAV-transduced (TGF-β/IGF-I versus TGF-β/*lac*Z, IGF-I/*lac*Z, and *lac*Z) hMSC aggregates (*n* = 3) were implanted over time (21 days) (Figure 4).

Enhanced chondrogenic differentiation was observed by higher SOX9 expression in the TGF-β/IGF-I versus *lac*Z, TGF-β/*lac*Z, and IGF-I/*lac*Z OCDs (6.4-, 3.1-, and 3.7-fold difference, *p* ≤ 0.014), in the TGF-β/*lac*Z versus *lac*Z and IGF-I/*lac*Z OCDs (2.1- and 1.7-fold difference, respectively, *p* ≤ 0.042), and in the IGF-I/*lac*Z versus *lac*Z OCDs (1.7-fold difference, *p* ≤ 0.001) (Figure 4). Enhanced chondrogenic COL2A1 expression was also seen in the TGF-β/IGF-I versus *lac*Z, TGF-β/*lac*Z, and IGF-I/*lac*Z OCDs (6.1-, 2.7-, and 5.3-fold difference, *p* = 0.057, *p* = 0.032, and *p* ≤ 0.001, respectively), in the TGF-β/*lac*Z versus *lac*Z and IGF-I/*lac*Z OCDs (2.2- and 2-fold difference, respectively, *p* ≥ 0.099), and in the IGF-I/*lac*Z versus *lac*Z OCDs (1.1-fold difference, *p* = 0.118) (Figure 4). Higher chondrogenic ACAN expression was also evidenced in the TGF-β/IGF-I versus *lac*Z, TGF-β/*lac*Z, and IGF-I/*lac*Z OCDs (6.4-, 3-, and 4.1-fold difference, *p* = 0.012, *p* = 0.066, and *p* = 0.054, respectively), in the TGF-β/*lac*Z versus *lac*Z and IGF-I/*lac*Z OCDs (2.2- and 1.4-fold difference, *p* = 0.007 and *p* = 0.186, respectively), and in the IGF-I/*lac*Z versus lacZ OCDs (1.6-fold difference, *p* = 0.083) (Figure 4).

Restricted osteogenic differentiation was seen by lower COL1A1 expression in the TGF-β/IGF-I versus *lac*Z, TGF-β/*lac*Z, and IGF-I/*lac*Z OCDs (1.6-, 1.3-, and 1.5-fold difference, respectively, *p* ≤ 0.001), in the TGF-β/*lac*Z versus *lacZ* and IGF-I/*lac*Z OCDs (1.2-fold difference, *p* ≤ 0.036), and in the IGF-I/*lac*Z versus lacZ OCDs (1.1-fold difference, *p* = 0.002) (Figure 4).

Reduced hypertrophic differentiation was noted by lower COL10A1 expression in the TGF-β/IGF-I versus *lac*Z, TGF-β/*lac*Z, and IGF-I/*lac*Z OCDs (1.4-, 1.3-, and 1.4-fold difference, *p* = 0.002, *p* = 0.069, and *p* = 0.018, respectively) and in the TGF-β/*lac*Z versus IGF-I/*lac*Z and *lac*Z OCDs (1.1-fold difference, *p* ≥ 0.181) while no difference was reported between the IGF-I/*lac*Z and *lac*Z OCDs (*p* = 0.440) (Figure 4).

Decreased catabolic processes were also observed by lower MMP13 expression in the TGF-β/IGF-I versus *lac*Z, TGF-β/*lac*Z, and IGF-I/*lac*Z OCDs (16.7-, 1.4-, and 1.7-fold difference, *p* ≤ 0.005), in the TGF-β/*lac*Z versus IGF-I/*lac*Z and *lac*Z OCDs (1.3- and 12.5-fold difference, *p* ≤ 0.001), and in the IGF-I/*lac*Z and *lac*Z OCDs (10-fold difference, *p* ≤ 0.001) (Figure 4).

## 4. Discussion

Cell-based strategies using the application of progenitor cells to focal cartilage defects may be of strong therapeutic value to enhance the intrinsic healing processes in sites of cartilage damage [7], especially following modification of such cellular platforms by gene therapy procedures that aim at stably triggering their chondroreparative activities [8,9]. Here, we tested the ability of the clinically adapted rAAV gene transfer vectors to co-deliver the highly chondrogenic TGF-β and IGF-I growth factors [11,15,16] to hMSCs for implantation in a model of human OCD that provides a natural environment similar to that noted in patients in vivo [20,21] as a means to repopulate the defects upon stimulation of the local healing processes relative to independent gene treatments and control (reporter gene vector) condition.

The data first indicate that rAAV is capable of successfully promoting the concomitant, sustained overexpression of TGF-β with IGF-I in hMSCs transplanted in human OCDs over the entire period of evaluation (at least 21 days) relative to single and reporter gene treatments, in agreement with the ability of rAAV to transduce and be maintained in such targets [11,13,14,15,16], especially in a simultaneous fashion [17]. Of note, the amounts of each respective growth factor produced in the OCDs were significantly higher upon the application of hMSCs co-transduced with TGF-β/IGF-I relative to single, independent candidate gene treatments at similar vector doses, suggesting a possible reciprocal regulation of growth factor expression as previously noted in articular chondrocytes [25]. The production levels of TGF-β and IGF-I raised from implanted TGF-β/*lac*Z- or IGF-I/*lac*Z-treated hMSCs were comparable to those measured in our previous work using solely rAAV-hTGF-β or rAAV-hIGF-I in hMSCs in vitro at equivalent MOIs (~ 70–300 pg/mL at MOI = 8) [15,16] or the current combination in settings in vitro aiming at directly rejuvenating the cells (~ 50–100 pg/mL at MOI = 8) [18]. Interestingly, transgene (co)-expression was also noted in the cartilage surrounding the defects where the respective candidate treatments were applied, showing the potentially beneficial production of the growth factors via rAAV-modified, transplanted cells at a distance. It remains to be seen whether too high and/or prolonged levels of growth factor co-overexpression may have a detrimental impact on tissue repair in this model system or in vivo—however, such an issue might be addressed by applying various (lower) vector doses or using regulatable (tetracycline-controlled) or tissue-specific promoters (*sox9*, type-II collagen) [26] instead of the strong CMV-IE regulatory element. While no immune responses were expected in such a model ex vivo as it lacks the full spectrum of immune components, they may occur in vivo [27] although to a low extent as rAAV has been reported for its absence of deleterious effects when directly applied to focal defects in vivo [28].

The results next show that significant, prolonged TGF-β/IGF-I co-overexpression from co-transduced, implanted hMSCs triggered the biological and chondrodifferentiation activities in human OCDs over time (cell proliferation, proteoglycan/type-II collagen deposition) more effectively than when using each individual candidate gene treatment and versus control (*lac*Z) condition, as a possible growth factor intercooperation [25] and concordant with the properties of TGF-β and IGF-I [5,6,11,29] and with our work using the vectors independently [15,16] or simultaneously in a direct transfer approach [18]. Such effects were also observed in the cartilage surrounding the defects, likely due to the expression of the candidate transgenes at a distance and/or to the paracrine effects of the factors secreted. A degree of variability was noted between samples, reflecting the different features and background of the individual donors, but in good agreement with previous observations by Mackay et al. [6] who reported similar discrepancies between human donors. Of note, as the gene expression profiles were tested in bone-free OCD-implant systems due to the limited size of the cell-confined aggregates, the contribution of the implants to such profiles remains to be determined [20] versus a possible impact of the (slightly less active) chondrocytes in the surrounding cartilage. Despite such results, integration between the implant and the host cartilage and self-repair (even for a small, 1-mm lesion) did not fully occur, in agreement with our previous observations using such a model system [20], an outcome that might be achieved at much longer time points [30,31].

Finally, the data demonstrate the ability of combined rAAV-mediated TGF-β/IGF-I co-overexpression in implanted hMSCs to prevent undesirable hypertrophic, terminal differentiation, and osteogenic differentiation as well as catabolic processes over time in treated OCDs relative to single candidate gene treatments and the control *lacZ* condition, probably due to higher levels of SOX9 expression, a transcription factor known for its anti-hypertrophic and anti-catabolic activities [14,32], and in agreement with our findings using a direct approach [18]. This is in contrast with our previous findings when applying either rAAV-hTGF-β or rAAV-hIGF-I to hMSCs in vitro [15,16] yet in these studies, a higher MOI was provided to the cells (MOI = 20 while we used an MOI = 8 here, i.e., a 2.5-fold difference), showing that the optimal treatment will be a function of the vector doses applied. Here also, these effects were seen in the cartilage surrounding the defects, again probably resulting from the expression of the transgenes at a distance and/or to paracrine effects of the products.

## 5. Conclusions

The present findings show the benefits of cell- and gene-based therapy using multiple therapeutic rAAV gene transfer procedures in order to regenerate human articular cartilage lesions with an adapted phenotype in a natural environment. Work is ongoing to evaluate the potential benefits of this indirect regenerative approach in clinically relevant animal models of (orthotopic) cartilage defects in vivo and to evaluate possible immune responses [33,34]. Overall, the current data provide a basis for the future treatment of focal cartilage defects in human individuals using the patient’s self-reparative progenitor cells.

## Figures and Tables

**Table 1:** Transgene expression in human OCD supernatants following implantation of rAAV-transduced hMSC aggregates (day 21). The levels of TGF-β production are in pg/mL and those of IGF-I in pg/mL. Data are given as mean ± SD. Statistically significant relative to ^a^*lac*Z, ^b^TGF-β/*lac*Z, and ^c^IGF-I/*lac*Z; TGF-β^+^, TGF-β-positive; IGF-I^+^, and IGF-positive.

Assay	*lacZ*	TGF-β/*lacZ*	IGF-I/*lacZ*	TGF-β/IGF-I
TGF-β^+^	156 ± 74	333 ± 58 ^a,c^	158 ± 90	556 ± 70 ^a,b,c^
IGF-I^+^	53 ± 7	55 ± 8	71 ± 16 ^a,b^	73 ± 18 ^a,b^

**Table 2:** Histomorphometric analyses in human osteochondral defects OCDs following implantation of rAAV-transduced hMSC aggregates (day 21). TGF-β-positive (TGF-β^+^) and IGF-positive (IGF-I^+^) cells are in %. The cell densities are as cells/mm^2^. Stained (safranin O) and immunostained (type-II, -I, and -X collagen) sections were scored for uniformity and intensity according to modified Bern Score grading system [19] as: 0 (no staining), 1 (heterogenous and/or weak staining), 2 (homogeneous and/or moderate staining), 3 (homogeneous and/or intense staining) and 4 (very intense staining). Data are given as mean ± SD. Statistically significant relative to ^a^*lacZ*, ^b^TGF-β/*lac*Z, and ^c^IGF-I/*lac*Z.

Assay		*lacZ*	TGF-β/*lacZ*	IGF-I/*lacZ*	TGF-β/IGF-I
TGF-β^+^ cells	OCD	12 ± 2	77 ± 8 ^a,c^	12 ± 5	93 ± 1 ^a,b,c^
	surrounding cartilage	2 ± 1	62 ± 6 ^a,c^	3 ± 1	74 ± 6 ^a,b,c^
IGF-I^+^ cells	OCD	13 ± 3	18 ± 4	76 ± 3 ^a,b^	86 ± 2 ^a,b,c^
	surrounding cartilage	6 ± 2	15 ± 4	56 ± 3 ^a,b^	64 ± 5 ^a,b,c^
Cell densities	OCD	3467 ± 115	5767 ± 1156 ^a^	5608 ± 300 ^a^	11,308 ± 1556 ^a,b,c^
	surrounding cartilage	145 ± 21	238 ± 75 ^a,c^	168 ± 11	286 ± 80 ^a,b,c^
Safranin O	OCD	0.3 ± 0.4	2.1 ± 0.8^a,c^	0.1 ± 0.4	2.4 ± 0.7 ^a,c^
	surrounding cartilage	1.3 ± 0.5	2.6 ± 1.0 ^a^	1.9 ± 0.6 ^a^	3.8 ± 0.5 ^a,b,c^
Type-II collagen	OCD	1.0 ± 0.8	3.0 ± 0.1 ^a,c^	2.3 ± 0.5 ^a^	3.8 ± 0.5 ^a,b,c^
	surrounding cartilage	0.5 ± 0.1	2.5 ± 0.6 ^a,c^	1.8 ± 0.5 ^a^	3.0 ± 0.8 ^a,c^
Type-I collagen	OCD	3.1 ± 0.8	1.5 ± 0.7 ^a,c^	3.0 ± 0.5	1.1 ± 0.4 ^a,c^
	surrounding cartilage	2.9 ± 0.6	2.0 ± 0.8 ^a^	2.6 ± 0.7	1.5 ± 0.5 ^a,c^
Type-X collagen	OCD	3.0 ± 0.8	1.9 ± 1.1 ^a^	2.3 ± 0.9	1.3 ± 0.5 ^a^
	surrounding cartilage	2.9 ± 0.6	2.6 ± 0.5	2.1 ± 1.0	0.8 ± 0.7 ^a,b,c^

**Table 3:** Biological activities in human OCDs following implantation of rAAV-transduced hMSC aggregates (day 21). The DNA contents are in ng/μg total proteins, the proteoglycan contents in μg/mg total proteins, and the proteoglycan/DNA contents in μg/ng. Data are given as mean ± SD. Statistically significant relative to ^a^*lacZ*, ^b^TGF-β/*lacZ*, and ^c^IGF-I/*lacZ*.

Assay	*lacZ*	TGF-β/*lacZ*	IGF-I/*lacZ*	TGF-β/IGF-I
DNA	0.39 ± 0.26	0.45 ± 0.30	0.39 ± 0.25	0.52 ± 0.32 ^a,b,c^
Proteoglycans	12 ± 7	17 ± 12 ^a^	15 ± 8	22 ± 13 ^a,b,c^
Proteoglycans/DNA	0.11 ± 0.05	0.13 ± 0.07 ^a^	0.12 ± 0.05	0.15 ± 0.07 ^a,b,c^
